# Supplementary material for: Factors that influence patient preferences for virtual consultations in an orthopaedic rehabilitation setting: a qualitative study
Source: BMJ Open. 2021 Feb 25;11(2):e041038. doi: 10.1136/bmjopen-2020-041038 (PMC7908916; doi:10.1136/bmjopen-2020-041038)
Supplement: Supplementary data [file bmjopen-2020-041038supp001.pdf]

CONNECT

Care in Orthopaedics burdeN of treatmeNt Effectiveness of Communication Technology

CONNECT Project Patient Interview Schedule

Part 1 – Burden of Treatment

- **Impact on Patient:** how technology consultations influence the experience of living with illness and engagement with clinical care

What was life like before you got your condition?

How does your condition affect you with daily life?
- Family
- Friends
- Work
- Hobbies
- Day to day activities and routine

Does anyone support you to manage your condition?

How do you manage your condition?
- Routine stuff
- Managing exacerbations

What medical services do you interact with, what for?
- Regulararity?

How would using communication technology impact on how you manage your condition?

Connect Project Topic Guide, version 1, 14.10.18

Part 2 – Results of Phase 1

- **Skills:** what were needed, how were they gained, how were they enacted in practice.

What skills do you think you would need in order to use Communication technology for your [physio / OT]? (Is there any difference between the two?)

Do you have the skills now? How would you get them? How could the RNOH support you to get them?

Describe how you think communication technology use would look in reality

- **Clinical Interactions:** impact of technology consultations on clinical interactions

What is the relationship like with you and your clinician now? Would it be different using communication technology? What could you still do? What couldn't you do? How would this make you feel?

How would it be with someone different? What would be 'a good person'. What would be a 'bad person'.

- **Environment:** the location and resources required to engage with clinical rehabilitation

What would you physically need to use communication technology? Where would you get it from? Where would you like to get it from (ie self-sourced or hospital sourced)

Where would you use it from? What space would you need to achieve the objectives of the consulation?

- **Processes:** how technology consultations affect routine clinical practice

What has your journey been as an RNOH patient
(previous care, how they got referred, waiting time, experience of being a patient, dealing with other services?)

How would this change with using communication technology? Wat would be better? What would be worse?

Connect Project Topic Guide, version 1, 14.10.18

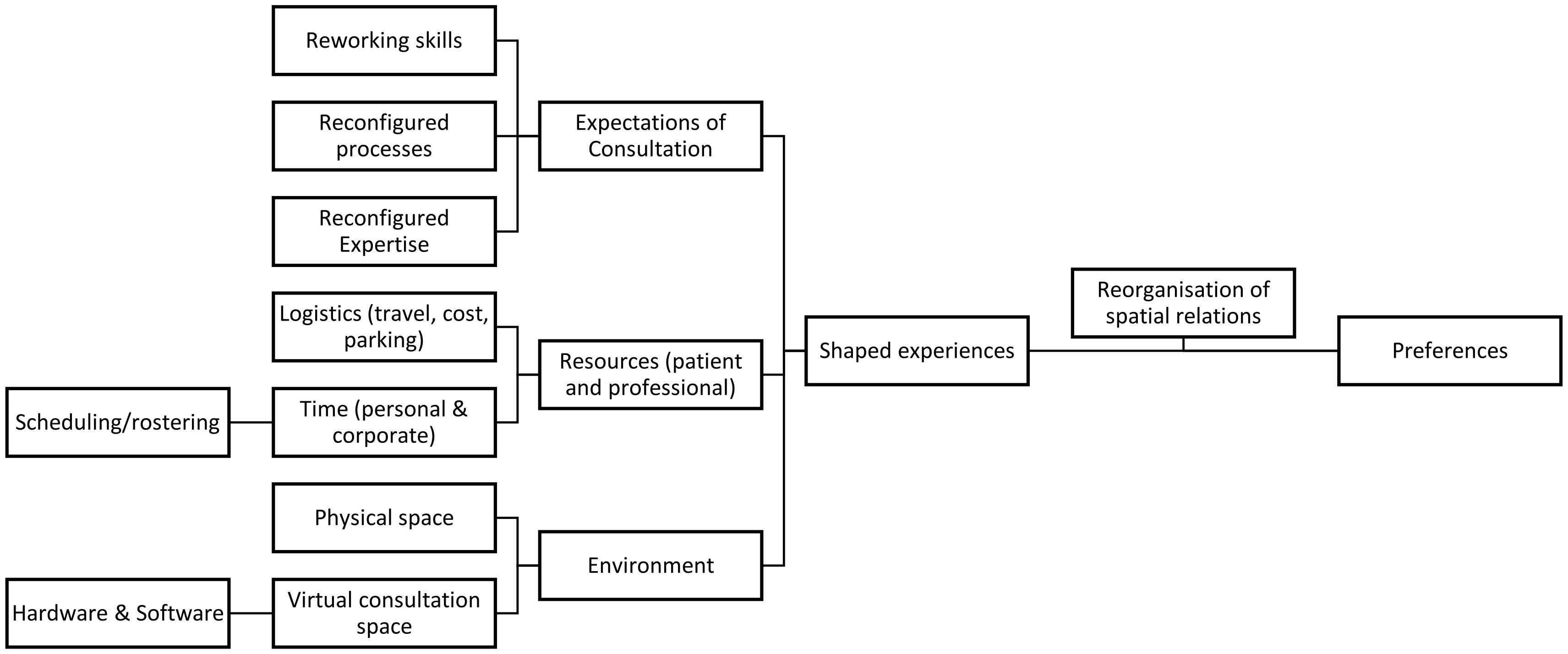

Connect Project Topic Guide, version 1, 14.10.18

Part 3 – DCE Design

- **Preferences:** the potential patients see for technology as an alternative to routine face to face care

In what situations would you be happy to use technology?

In what situations would you not be happy to use?

What would use look like for you at the RNOH?

What would we need to consider? From a personal perspective? From others' perspective?

Anything you would like to add that might help the research?

Connect Project Topic Guide, version 1, 14.10.18
