# Supplementary material for: Factors that influence patient preferences for virtual consultations in an orthopaedic rehabilitation setting: a qualitative study
Source: BMJ Open. 2021 Feb 25;11(2):e041038. doi: 10.1136/bmjopen-2020-041038 (PMC7908916; doi:10.1136/bmjopen-2020-041038)
Supplement: Supplementary data [file bmjopen-2020-041038supp002.pdf]

CONNECT Project Clinician Interview Schedule

Part 1 – Burden of Treatment

- **Impact on Patient:** how technology consultations influence the experience of living with illness and engagement with clinical care

Can you give me examples of how patients' conditions affect their life? eg
- Family
- Friends
- Work
- Hobbies
- Day to day activities and routine

Do your patients need support to manage their condition?

How do patients manage their condition?
- Routine stuff
- Managing exacerbations

What medical services do your patients interact with, what for?
- Regulararity?

How would using communication technology impact on how patients manage their conditions?

Gilbert AW, *et al. BMJ Open* 2021; 11:e041038. doi: 10.1136/bmjopen-2020-041038

Part 2 – Results of Phase 1

- **Skills:** what were needed, how were they gained, how were they enacted in practice.

What skills do you think patients would need in order to use Communication technology for your [physio / OT]? (Is there any difference between the two?)

Do your patients have the skills now? How would they get them? How could the RNOH support them to get them?

Describe how you think communication technology use would look in reality

- **Clinical Interactions:** impact of technology consultations on clinical interactions

Would it be different using communication technology? What could you still do? What couldn't you do? How would this make your patients feel?

How would it be with someone different? What would be 'a good person'. What would be a 'bad person'.

- **Environment:** the location and resources required to engage with clinical rehabilitation

What would you physically need to use communication technology? Where would patients get it from? Where would they like to get it from (ie self-sourced or hospital sourced)

Where would they use it from? What space would they need to achieve the objectives of the consultation?

- **Processes:** how technology consultations affect routine clinical practice

Think of your patients pathway….
(previous care, how they got referred, waiting time, experience of being a patient, dealing with other services?)

How would this change with using communication technology? What would be better? What would be worse?

CONNECT
Care in Orthopaedics burdeN of treatmeNt Effectiveness of Communication Technology

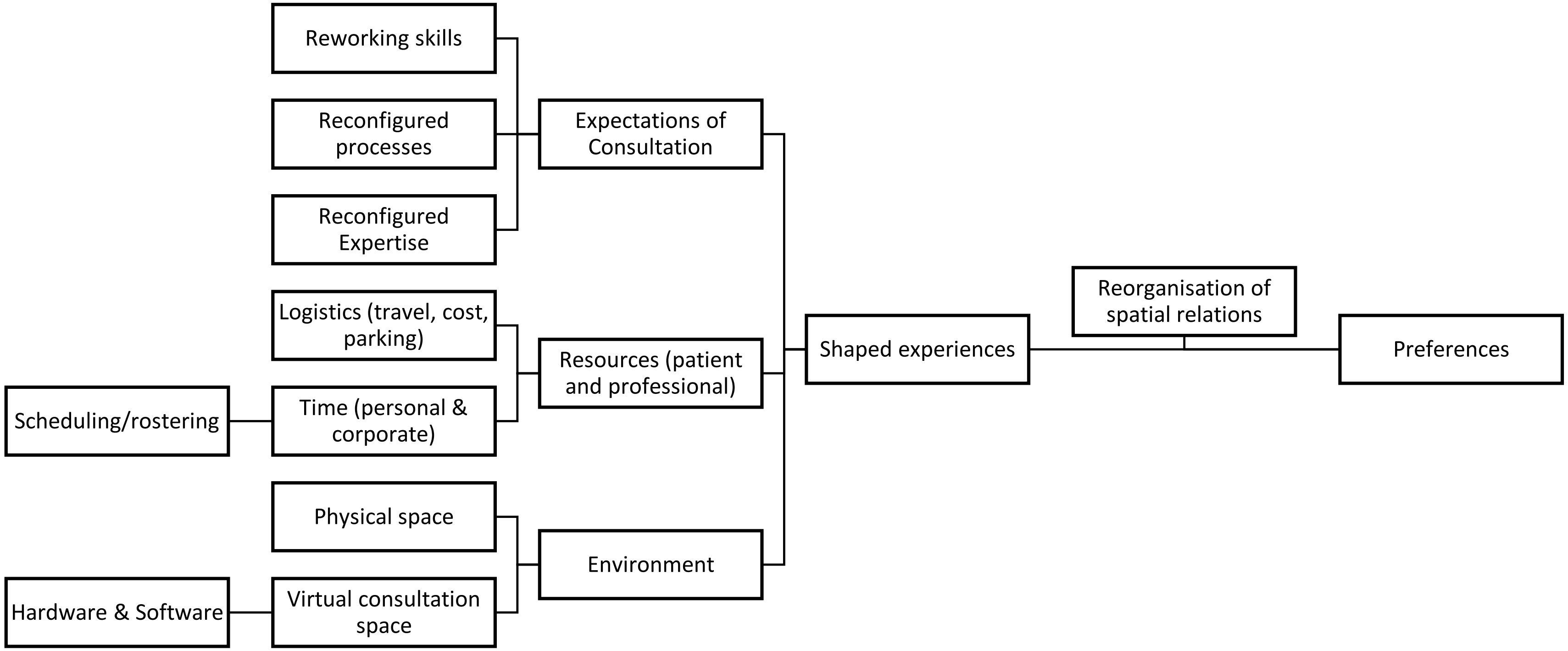

Connect Project Topic Guide (clinicians), version 1, 14.10.18

CONNECT

Care in Orthopaedics burdeN of treatmeNt Effectiveness of Communication Technology

Part 3 – DCE Design

- **Preferences:** the potential patients see for technology as an alternative to routine face to face care

In what situations would patiens be happy to use technology?

In what situations would patients not be happy to use?

What would use look like for you at the RNOH?

What would we need to consider? From a personal perspective? From others' perspective?

Anything you would like to add that might help the research?

Gilbert AW, *et al. BMJ Open* 2021; 11:e041038. doi: 10.1136/bmjopen-2020-041038
