# Supplementary material for: Factors that influence patient preferences for virtual consultations in an orthopaedic rehabilitation setting: a qualitative study
Source: BMJ Open. 2021 Feb 25;11(2):e041038. doi: 10.1136/bmjopen-2020-041038 (PMC7908916; doi:10.1136/bmjopen-2020-041038)
Supplement: Supplementary data [file bmjopen-2020-041038supp004.pdf]

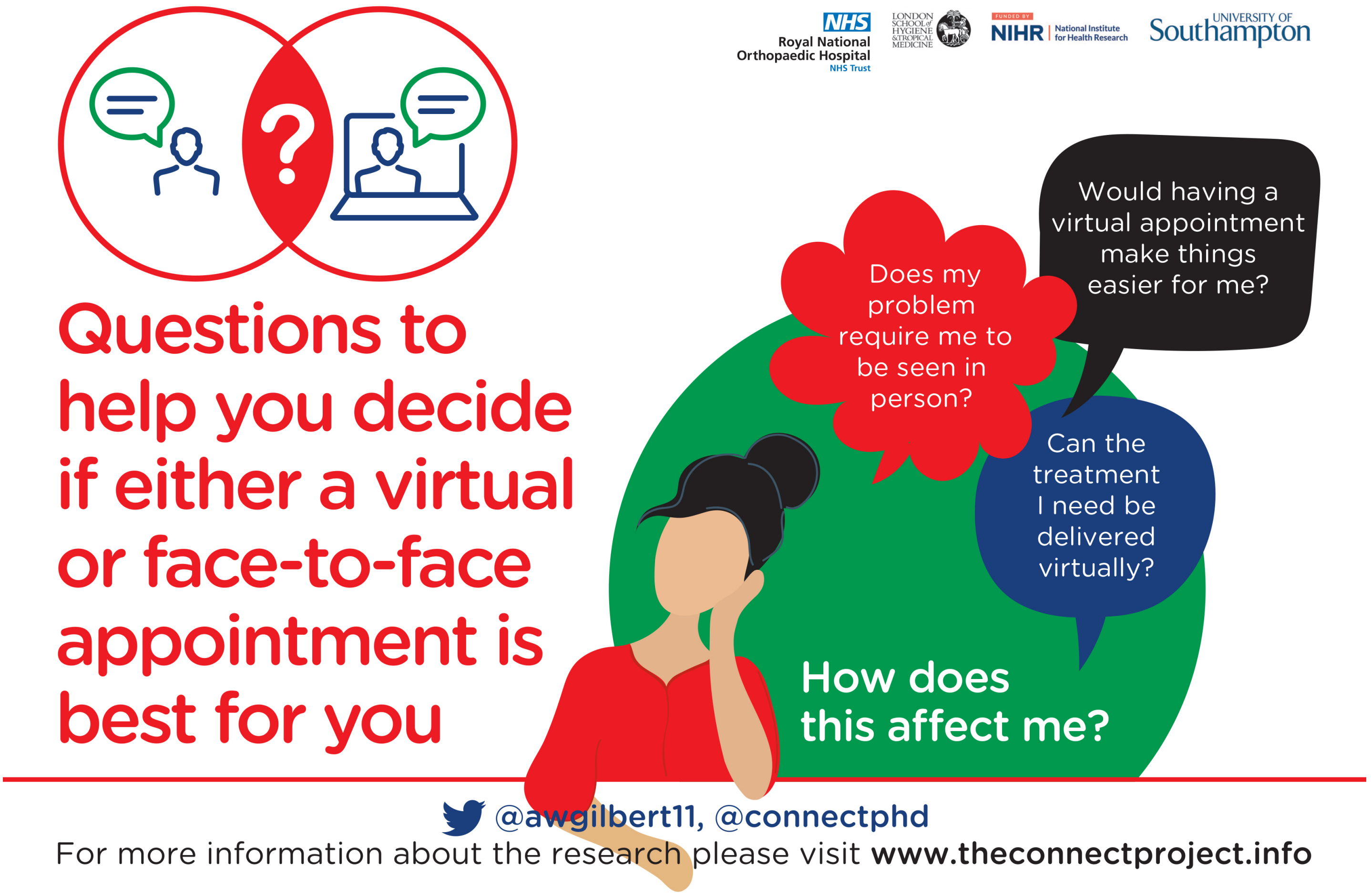
Royal National Orthopaedic Hospital NHS Trust
London School of Hygiene & Tropical Medicine
Funded by NIHR National Institute for Health Research
University of Southampton
Questions to help you decide if either a virtual or face-to-face appointment is best for you
Would having a virtual appointment make things easier for me?
Does my problem require me to be seen in person?
Can the treatment I need be delivered virtually?
How does this affect me?
@awgilbert11, @connectphd
For more information about the research please visit www.theconnectproject.info
