# Supplementary material for: Factors that influence patient preferences for virtual consultations in an orthopaedic rehabilitation setting: a qualitative study
Source: BMJ Open. 2021 Feb 25;11(2):e041038. doi: 10.1136/bmjopen-2020-041038 (PMC7908916; doi:10.1136/bmjopen-2020-041038)
Supplement: Supplementary data [file bmjopen-2020-041038supp006.pdf]

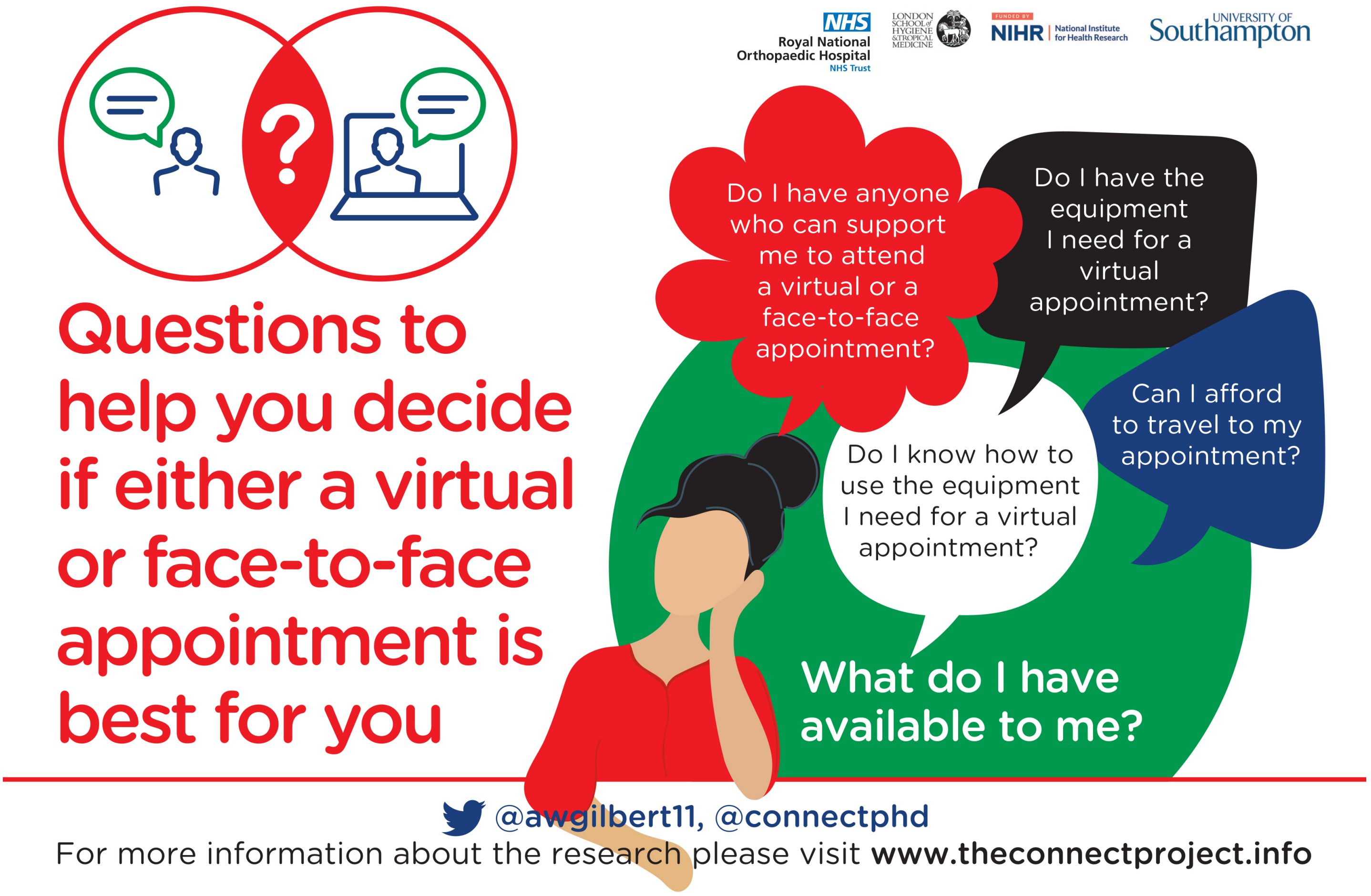
Royal National Orthopaedic Hospital NHS Trust
NIHR National Institute for Health Research
University of Southampton
Questions to help you decide if either a virtual or face-to-face appointment is best for you
Do I have anyone who can support me to attend a virtual or a face-to-face appointment?
Do I have the equipment I need for a virtual appointment?
Can I afford to travel to my appointment?
Do I know how to use the equipment I need for a virtual appointment?
What do I have available to me?
@awgilbert11, @connectphd
For more information about the research please visit www.theconnectproject.info
