# Supplementary material for: Factors that influence patient preferences for virtual consultations in an orthopaedic rehabilitation setting: a qualitative study
Source: BMJ Open. 2021 Feb 25;11(2):e041038. doi: 10.1136/bmjopen-2020-041038 (PMC7908916; doi:10.1136/bmjopen-2020-041038)
Supplement: Supplementary data [file bmjopen-2020-041038supp007.pdf]

Royal National Orthopaedic Hospital NHS Trust · London School of Hygiene & Tropical Medicine · Funded by NIHR National Institute for Health Research · University of Southampton

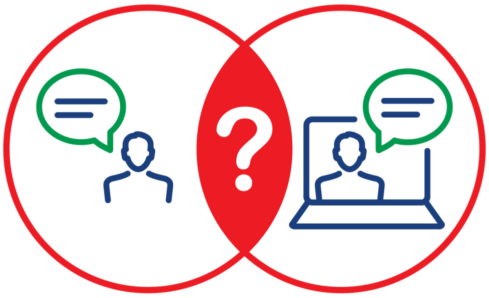

Questions to help you decide if either a virtual or face-to-face appointment is best for you

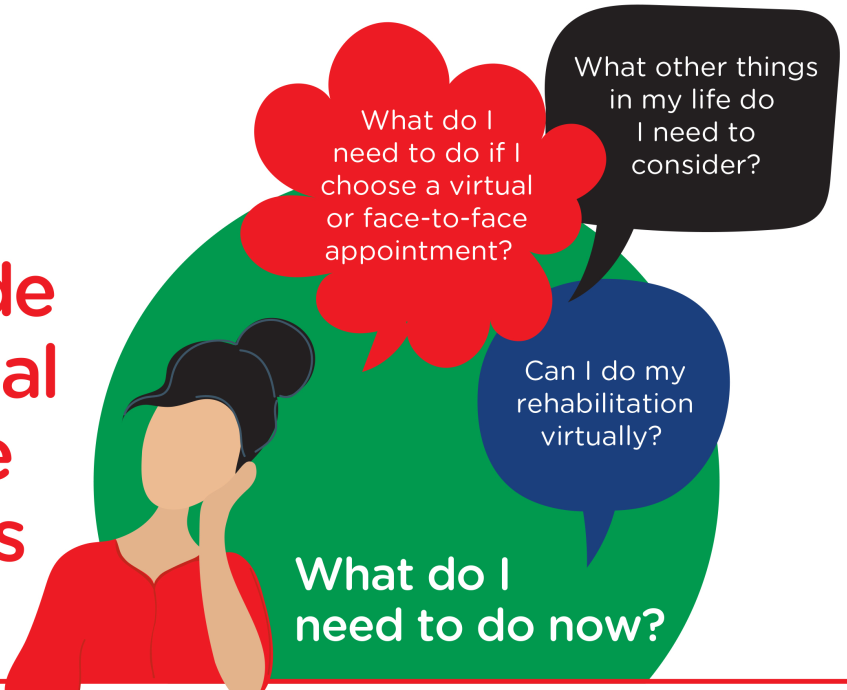

@awgilbert11, @connectphd

For more information about the research please visit **www.theconnectproject.info**
